# An Effective Mental Stress State Detection and Evaluation System Using Minimum Number of Frontal Brain Electrodes

**DOI:** 10.3390/diagnostics10050292

**Published:** 2020-05-09

**Authors:** Omneya Attallah

**Affiliations:** Department of Electronics and Communications, College of Engineering and Technology, Arab Academy for Science, Technology and Maritime Transport, Alexandria 1029, Egypt; o.attallah@aast.edu

**Keywords:** electroencephalogram (EEG), mental stress detection (MSD), machine learning, mental arithmetic

## Abstract

Currently, mental stress is a common social problem affecting people. Stress reduces human functionality during routine work and may lead to severe health defects. Detecting stress is important in education and industry to determine the efficiency of teaching, to improve education, and to reduce risks from human errors that might occur due to workers’ stressful situations. Therefore, the early detection of mental stress using machine learning (ML) techniques is essential to prevent illness and health problems, improve quality of education, and improve industrial safety. The human brain is the main target of mental stress. For this reason, an ML system is proposed which investigates electroencephalogram (EEG) signal for thirty-six participants. Extracting useful features is essential for an efficient mental stress detection (MSD) system. Thus, this framework introduces a hybrid feature-set that feeds five ML classifiers to detect stress and non-stress states, and classify stress levels. To produce a reliable, practical, and efficient MSD system with a reduced number of electrodes, the proposed MSD scheme investigates the electrodes placements on different sites on the scalp and selects that site which has the higher impact on the accuracy of the system. Principal Component analysis is employed also, to reduce the features extracted from such electrodes to lower model complexity, where the optimal number of principal components is examined using sequential forward procedure. Furthermore, it examines the minimum number of electrodes placed on the site which has greater impact on stress detection and evaluation. To test the effectiveness of the proposed system, the results are compared with other feature extraction methods shown in literature. They are also compared with state-of-the-art techniques recorded for stress detection. The highest accuracies achieved in this study are 99.9%(sd = 0.015) and 99.26% (sd = 0.08) for identifying stress and non-stress states, and distinguishing between stress levels, respectively, using only two frontal brain electrodes for detecting stress and non-stress, and three frontal electrodes for evaluating stress levels respectively. The results show that the proposed system is reliable as the sensitivity is 99.9(0.064), 98.35(0.27), specificity is 99.94(0.02), 99.6(0.05), precision is 99.94(0.06), 98.9(0.23), and the diagnostics odd ratio (DOR) is ≥ 100 for detecting stress and non-stress, and evaluating stress levels respectively. This shows that the proposed framework has compelling performance and can be employed for stress detection and evaluation in medical, educational and industrial fields. Finally, the results verified the efficiency and reliability of the proposed system in predicting stress and non-stress on new patients, as the accuracy achieved 98.48% (sd = 1.12), sensitivity = 97.78% (sd = 1.84), specificity = 97.75% (sd = 2.05), precision = 99.26% (sd = 0.67), and DOR ≥ 100 using only two frontal electrodes.

## 1. Introduction

Mental stress is the human body’s response to exposed psychosocial or physical situations. It affects people all over the world regardless of their age, gender, or occupation. This is because of the increasing work difficulties, rising pressure, and demanding daily activities that people face every day [1]. Currently, mental stress is considered the main cause of several health problems. These problems include heart attacks, strokes, nervousness, depression, post-traumatic stress disorder (PTSD), and immunological disorders. Stress can also influence brain activity and structure [2]. Therefore, early detection of stress is essential to prevent illness and reduce the chances of clinical brain damage and other health problems.

Detection and the evaluation of mental stress is essential as well in fields such as education and industry. In educational e-learning settings, stress may be a major factor that affects students’ performance in exams. Stress level may increase with unhealthy examination schemes in higher education. In such systems, students can be rated on their performance based on limited hours only. Accordingly, their grades may not represent their real knowledge and intelligence but rather their ability to cope with exam induced stress [3]. Additionally, in an offline educational setting, the frequent evaluation of student’s mental state can be used to define the speed of teaching and improve the outcome of education [4]. For industrial security, recognizing hazards that occurs due to human mistakes is essential. This is because insecure and careless manners of workers and absence of safety measures are the major reasons for human-caused problems. Such factors include lack of sufficient sleep, poor diet, physical deficiencies, and fatigue, which can lead a person into a stressful situation.

Common methods to measure stress include questionnaires, where the mental effort that applicants put into a task is evaluated [5]. However, such methods can be subjective, i.e., depend on the personal opinion of the applicants, based on psychophysiological [6] or personal measures [7]. Therefore, these methods are not accurate enough due to individuals’ inconsistencies. Moreover, this process becomes challenging when the number of individuals to be evaluated increases in real time. Thus, automated stress detection algorithms that can correctly recognize and assess stress even with a large number of subjects are important in identifying stress factors and facilitating stress management. These approaches embrace the use of portable devices such as mobiles, remotes, or wearable sensing devices to gather physiological signals, such as electrocardiograms (ECGs), near-infrared spectroscopy (NIRS), near-infrared spectroscopy (NIRS), functional magnetic resonance imaging (fMRI), electro-corticography (ECoG) or electroencephalograms (EEGs) [8,9]. 

Recently, brain activity measurement has been verified as an efficient method in the imaging of emotional stress changes [10]. NIRS and fMRI measure brain activity using blood in the brain. The strength of fMRI is its ability to capture signals in the brain with an outstanding resolution, nevertheless, the measurements are deferred until the state of the brain changes. In contrast, NIRS has the ability to describe only the state of the brain and the signal is eventually captured through blood flow. The EEG and ECoG waves measure the brain signals as well. Regardless of the ability of the ECoG to measure long-bandwidth signals, a surgical procedure is needed to insert electrodes on the skull to detect these signals. EEG is measured non-invasively; it employs a process that requires wearing a helmet. It measures signals from the scalp rather than from the brain itself [3]. Therefore, EEG is preferred over other methods. EEG is commonly used to measure stress [11,12,13,14] due to the increasing availability of EEG systems as low-cost wearable devices [15]. Additionally, it has a comparatively greater temporal resolution and it can visualize fast and energetically varying brainwave patterns in complex stress scenarios [9] Therefore, in this study, EEG is used. The main purpose of this paper is to identify the mental state of a person by analyzing the EEG signals. EEG is a noisy signal. Medical signal processing (MSP) techniques play an important role in removing this noise and retaining only the frequency bands containing informative data that describe mental stress. MSP can also extract useful features from EEG signals. Many feature extraction techniques are used for the analysis of EEG signals. Some of these methods include; Hjorth parameters [16,17], power spectral density (PSD) [18,19], common spatial patterns (CSPs) [9,14], statistical-based [20,21], and wavelet-based methods [22,23]. Features extracted using feature extraction techniques are fed into a classification model to either classify stress, non-stress, or stress levels. Classification based on machine learning techniques has the ability to accurately classify stress states. This can assist doctors to understand the signals well, make an accurate diagnosis and provide appropriate treatment [1,8].

Recently, many studies have validated the associations between EEG pattern and emotional stress state [24]. These studies examine stress, non-stress, and stress levels from EEG signals. The authors in [25] proposed a framework that applies the concepts of network physiology and information theory to extract valuable features. A random forest (RF) classifier was constructed to distinguish between stress and non-stress situations. Mahajan, in [18], proposed two feature-sets to classify stress using an artificial neural network (ANN). In [6], a threshold-based method was introduced to detect stress. The number of peaks of the theta band of an EEG signal was counted. If this count exceeded the threshold, then it was considered a stressful state. The authors of [14] used CSP as a feature extractor. A linear discriminant classifier (LDA) was used to distinguish between stress and non-stress. A support vector machine classifier was employed in [26] to differentiate between stress levels using power spectral density (PSD) features. PSD features were also used in [19] to discriminate between stress and non-stress conditions using an ANN classifier. In the research articles [20,27], we assessed stress levels using a support vector machine (SVM) classifier. In [28], an SVM classifier was also used to classify the mental stress state of individuals based on the changes in EEG power spectral density, especially in the theta and alpha bands, where the average classification accuracy reached 79% and 78%, respectively. The authors in [4] employed a single channel EEG device to examine the use of frontal EEG to determine stress levels. An SVM classifier was employed to evaluate stress levels and the accuracy achieved was in the range of 65%–75%. The authors in [10] employed classical machine learning classifiers with a feature selection approach to detect stress levels. In [11], a hybrid feature pool was constructed to recognize stress and non-stress using KNN. Donghoo et al. in [29] proposed a genetic algorithm (GA)-based feature selection algorithm and used and *k*-nearest neighbor (KNN) classifier to identify stress and non-stress. Fares et al. in [30,31] proposed an MSD to detect stress using an SVM classifier. Most of the previous techniques used a large number of electrodes to construct their MSD systems. The performance of such systems was not sufficient enough for real applications. They also lacked efficient feature extraction, selection, or reduction techniques to enhance the performance of a subsequent classifier. 

Other MSD systems were constructed based on deep learning techniques such as Li et al. [24], who used a fused deep learning architecture to extract discriminative spatial–temporal EEG features for detecting emotional stress. Hefron et al. [32] presented a novel convolutional recurrent neural classifier by means of multipath subnetworks for detecting stress. Kuanar et al. [33] built a recurrent neural network algorithm to complete the cognitive analysis of EEG signals. Such methods based on deep learning have several drawbacks. First of all, for efficient performance using deep learning, deep networks usually require large amounts of data for training. Furthermore, deep features extracted from deep networks are commonly not separable, are highly correlated, and have a huge feature dimensional space. Additionally, the current methods do not consider any significance to the selection of appropriate features from specific domains for deeper fundamental analysis. A summary of the recent related techniques from literature that are close to the proposed MSD system are presented in Table 1.

The key aim of this paper is to construct a portable real-time EEG-based mental training neuro-feedback system to identify and evaluate stress levels efficiently in real-time. Thus, a new MSD system is proposed to classify stress, non-stress, and stress levels. Relative features are the key factor in a powerful MSD system. Therefore, the proposed technique presents a hybrid feature subset for MSD. Five classifiers are used to detect stress situations, then the stress level is assessed and sorted into low and high. In order to generate an efficient MSD system with a lower number of channels, first, the proposed MSD scheme explores the placements of electrodes on different sites on the skull and selects the location which has the highest influence on the accuracy of the system. Furthermore, Principal Component Analysis (PCA) feature reduction is applied in a sequential forward search to select the optimal number of principal components and reduce the dimension of feature space, which usually enhances the performance of the subsequent classifier. In addition, in order to make the proposed MSD system more portable and movable and easier to set up, the number of electrodes is further reduced by selecting the minimum number of channels from the site which has the greater impact on the stress detection rates. To prove the efficiency of the proposed method, the results are compared with three feature-sets proposed in literature. They are also compared with the state-of-the-art techniques.

## 2. Materials and Methods

### 2.1. Participants

Initially, sixty-six subjects (age: µ = 18.6, σ = 0.87) participated in the data collection procedure, and 47 of them were women and 19 were men. According to EEG visual examination by an expert neurophysiologist, thirty of them were omitted from the data collection process because of poor EEG quality (extreme amount of myographic and oculographic artifacts), so the final sample size is thirty-six participants [40]. Details of the participants can be found in Table 2. Subjects were qualified to register in the study if they had no medical signs of cognitive or mental deficiency, verbal or non-verbal education incapacities, and had usual or corrected-to-usual visual perception, and normal color sight. Exclusion criteria were the use of medication or alcohol addiction, psychoactive drug, and neurological or illnesses.

### 2.2. EEG Collection Procedure

Mental arithmetic evaluation is commonly used as a standard stressor [41]. Therefore, in this paper, mental arithmetic tasks are used. The tasks consist of counting during the relaxed state (non-stress) and serial subtraction of two numbers during the stress state. Every serial subtraction trial begins with verbally subtracting 4 numbers from 2. Serial subtraction during 15 min is known as psychosocial stress [42], and it was used in [40]. In this manner, the design of the data collection procedure in [40] required exhaustive cognitive activity from the engaged individuals. This strenuous mental load leads to a variation in the emotional background once the individual involves more effort to solve tasks. Depending on quantity of arithmetic operations per minute, participants were grouped into two classes. The first class are called “high stress” and contains participants who performed the arithmetic tasks with difficulty and make more effort to perform the arithmetic tasks. The other class is called “low stress” and consists of participants who managed the arithmetic tasks without difficulty and no excess effort. Thus in this study, two mental stress states are considered.

Subjects were asked to sit on a comfortably reclined armchair in a dark soundproof hall. Before starting the experiment, they were asked to relax throughout the resting state (non-stress state) and informed about the arithmetic task. Subjects were instructed to count accurately and quickly for 3 min without talking or moving in the pace they chose. Afterwards, they performed serial subtraction and the EEG was recorded for 1 min during this state (stress condition). 

**Figure 1:**
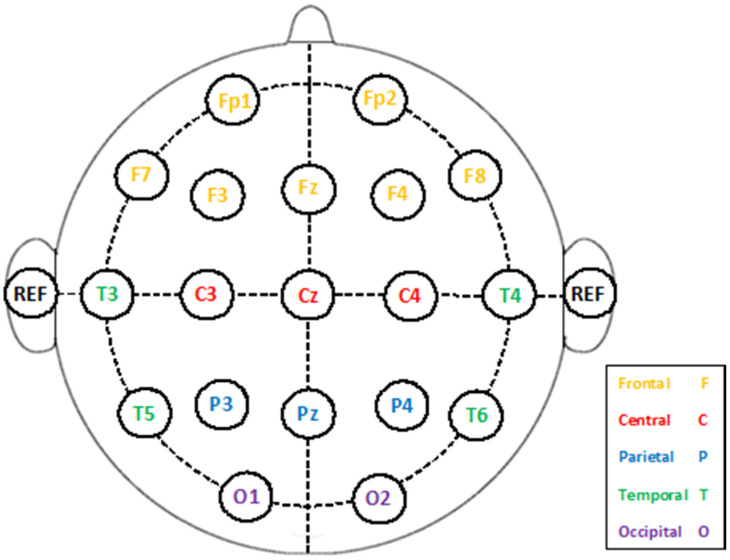
Electrodes placement on the scalp.

The EEG dataset was acquired using the Neurocom monopolar EEG 23 channel system. Silver/silver chloride electrodes were placed on the scalp to record the data according to the International 10/20 scheme. Electrodes were inserted on different locations on the scalp. These sites comprise temporal (T3, T4, T5, T6), frontal (Fp1, Fp2), frontal (F3, F4, Fz, F7, F8), central (C3, C4, Cz), occipital (O1, O2), and parietal (P3, P4, Pz), according to the International 10/20 system. Ear electrodes were used as a reference. Electrode sites on the scalp are shown in Figure 1.

### 2.3. Proposed Mental Stress Detection System

**Figure 2:**
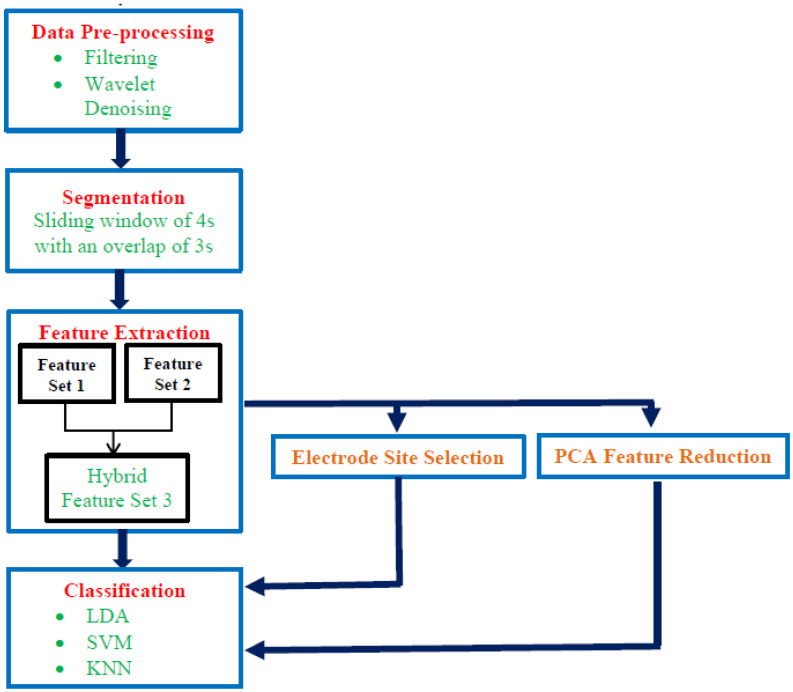
Block diagram of the proposed system.

In this paper, a new system is proposed to detect stress states and classify stress levels. The proposed system consists of five steps as shown in Figure 2. Initially, the data are preprocessed, and noise and artifact removal constitutes the first step. Subsequently, the data are segmented into frames by a sliding window. Afterwards, valuable features are extracted using time and frequency feature extraction techniques to yield two feature-sets which are combined later to form a hybrid feature-set. Then, a feature reduction step is used to reduce the dimension of the combined feature-set. Finally, five machine classifiers are used to detect stress and evaluate stress levels. The proposed MSD system is composed of three experiments. In the first experiment, each of the two feature-sets are used individually to construct the MSD system, and then combined to form a hybrid feature-set to examine the influence of combining time and frequency features. In experiment two, to reduce the number of channels employed to construct the MSD system and enhance the effectiveness of the proposed approach, the electrodes are placed on different sites on the skull and the location which has the greatest influence on the accuracy of the system is chosen. Finally, in experiment three, the PCA feature reduction method is adopted to choose the optimum number of principal components in sequential forward search and reduce the dimension of feature space. Reducing feature space commonly improves the performance of an MSD system.

#### 2.3.1. Data Preprocessing

An EEG signal is usually noisy due to power line interference, electromyography (EMG), electrocardiography (ECG), and subject movement, etc. In order to reduce noise and artifacts, a high-pass filter with 0.5 Hz cut-off frequency, low-pass filter with 500 Hz cut-off frequency and a notch filter of 50 Hz were used. The filters used were Butterworth IIR filters of order 4. The distortion of the filters was handled using forward–reverse filtering. In addition, a de-noising method based on multilevel wavelets decomposition was employed [43]. The number of wavelet levels was 5, the mother wavelet was Symlets. The number of vanishing moments was 4. The signals are further smoothed using a Savitzky–Golay filter [44].

#### 2.3.2. Segmentation

EEG pre-processed signals are segmented into 4-s segments with a sliding window of 1 s as an increment step. The same segmentation procedure as [12] is followed which stated that a window size of 4 s is suitable and common for classification using a mental arithmetic task. Each of these segments is considered as a single trial. Thus, each trial will have a size of N × T, where N = 19 is the number of channels and T = 4 corresponds to 4 s of one segmented frame sampled at 500 Hz. All trials will be used later in the feature extraction and classification steps.

#### 2.3.3. Feature Extraction

To construct a machine learning classifier, useful features are needed to be extracted from the EEG segmented data. For this purpose, for each trial of an EEG signal, two subsets of features are extracted.

Previous studies have demonstrated that to detect emotional activities like stress with higher accuracy, it is preferable to extract features from the frequency domain [45]. Other studies mentioned that fusing time and frequency features has better impact for the detection rates of stress [46,47]. Therefore, in this paper, we proposed using time and frequency features and studied the impact of fusing these features. The Feature-Set 1 is a frequency feature-set which includes features which were used in [48]. These features are the median frequency (MDF), modified frequency mean (MFMD) features, and spectral moments (SM). MDF computes the median normalized angular frequency. SM calculates three power spectral moments from each EEG segment corresponding to root squared zero, second and forth order moment. MDMD it is the frequency at which the spectrum is split into two sections with equivalent amplitude. In other words, it determines the median amplitude spectrum in each segment calculated using Fourier transform. Feature-Set 2 consists of the following features; the root mean square (RMS) amplitude of the signal which was used in [49,50] and a sixth order autoregressive (AR) model coefficients which was used in [51,52]. AR uses each sample of EEG segment to describe it as a linear fusion with the preceding samples plus a white noise error term. AR calculates coefficients of the model depending on the order chosen. Such coefficients are considered as features [53]. Feature-Sets 1 and 2 are combined together to form a hybrid subset called Feature-Subset 3. The equations representing the features are shown below.

(1) $$MDF=\frac{1}{2}\overset{M}{\underset{j=1}{\sum }}PSD_{j}$$

(2) $$MFMD=\frac{1}{2}\overset{M}{\underset{k}{\sum }}A_{k}\;\;\;\;\;\;$$

where *M* is the size of the power spectrum density, and *PSDj* is the *j*th line of the power spectrum density; *A_k_* is the EEG amplitude spectrum at frequency index *k*.

(3) $$m_{o}=\sqrt{\overset{N-1}{\underset{i}{\sum }}x_{i}^{2}}$$

(4) $$\bar{m_{2}}=\sqrt{\overset{N-1}{\underset{k=0}{\sum }}k^{2}}P_{k}\;P_{k}=\frac{1}{N}\overset{N-1}{\underset{k=0}{\sum }}\left|X_{k}X_{k}^{*}\right|$$

(5) $$\bar{m_{4}}=\sqrt{\overset{N-1}{\underset{k=0}{\sum }}k^{4}}P_{k}\;P_{k}=\frac{1}{N}\overset{N-1}{\underset{k=0}{\sum }}\left|X_{k}X_{k}^{*}\right|$$

where a sampled version of the EEG segment is denoted as *x_i_*, with *i* = 1,2,…*N*, of length *N*, $\bar{m_{o}}$, $\bar{m_{2}}$, and $\bar{m_{4}}$ are the root squared zero, second and forth order moment. The discrete frequency transform of an EEG segment can be expressed as a function of frequency *X_k_*. *P_k_* is the phase-excluded power spectrum which is equivalent to the result of a multiplication of *X_k_* by its conjugate X_k_* divided by *N*, and *k* is the frequency index.

(6) $$RMS=\sqrt{\frac{1}{N}\overset{N}{\underset{i=1}{\sum }}x_{i}^{2}}$$

(7) $$AR=\overset{D}{\underset{d=1}{\sum }}a_{d}x_{i-d}+e$$

where *a_d_* is AR coefficients, *e* is white noise or error sequence, and *D* is the order of AR model, *d* is the order of the coefficient *d* = 1,2,…..*D*.

#### 2.3.4. Feature Reduction

The hybrid Feature-Set 3 extracted in the previous step is of high dimensional space, and for this reason the feature reduction process is required to lower the dimension of the feature space, reduce the cost of computation of the training process, decrease the complication of the MSD system, improve the efficiency of stress detection process, and produce a reliable portable real time MSD system. Principal Component Analysis (PCA) is a common feature reduction technique used to reduce the size of Feature-Set 3 by applying a covariance analysis. PCA shrinks the number of features in Feature-Set 3 to a lower number of principal components [54]. Such principle components convey the variance of the features in Feature-Set 3. The steps of PCA technique used in to reduce Feature-Set 3 are as follows; first, we calculate the covariance matrix of Feature-Set 3. Afterwards, we determine the eigenvectors and the eigenvalues of the covariance matrix. Next, we select the number of the principal components using a sequential forward procedure. Finally, a reduced feature-set is produced.

#### 2.3.5. Classification

The three feature-sets generated in the feature extracted phase are used to construct five well-known classification models. These models are linear discriminate analysis (LDA), *k*-nearest neighbor (KNN), linear and cubic support vector machine (SVM) classifiers, and random forest classifiers. The distance metric that is used for the KNN is Euclidian and the number of neighbors (K) is equal to 1. These classification models are first used to detect stress and non-stress states. Next, they are used to distinguish between two stress levels (low and high stress levels). All models are tested using 5-fold cross-validation.

### 2.4. Performance Evaluation

Several metrics are used to evaluate the performance of the proposed system. These metrics are the classification accuracy (CA), sensitivity, specificity, Goodness Index (G), precision, Matthew correlation coefficient, diagnostic odds ratio (DOR), and receiver operating characteristic (ROC) analysis.

(8) $$CA=\frac{TP+TN}{TN+FP+FN+TP}$$

(9) $$Sensitivity=\frac{TP}{TP+FN}$$

(10) $$Specificity=\frac{TN}{TN+FP}$$

(11) $$GoodnessIndex=\sqrt{{\left(1-Sensitivity\right)}^{2}+{\left(1-Specificity\right)}^{2}}$$

(12) $$Precision=\frac{TP}{TP+FP}$$

(13) $$MCC=\frac{TP\times TN-FP\times FN}{\sqrt{\left(TP+FP\right)\left(TP+FN\right)\left(TN+FP\right)\left(TN+FN\right)}}$$

(14) $$DOR=\frac{TP\times TN}{FP\times FN}$$

where *TP* is the true positive, which is the number of positive class instances that are correctly classified. *TN* is the true negative, which is the number of negative class instances that are correctly classified. *FP* is the false positive, which is the number of negative class instances that are incorrectly classified as positive class, and *FN* is the false negative, which is the number of positive class instances that are incorrectly classified as negative class.

## 3. Results

**Figure 3:**
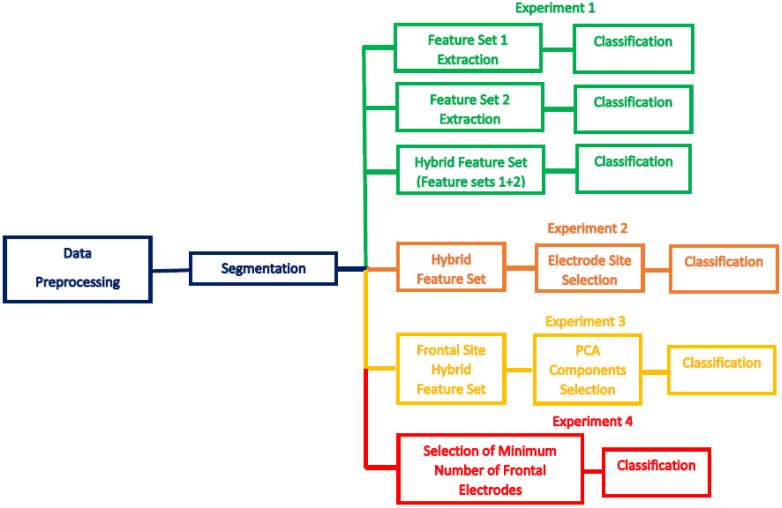
A workflow to explain the three experiments of the proposed MSD system.

The main objective of this study is to build a portable real-time EEG-based system to detect stress states and distinguish between stress levels. The mental arithmetic test is a popular stress inducer, and so is used in this study. This study presents a new MSD system which consists of four phases, or experiments. Phase one—two feature extraction methods are used to extract valuable features from segmented EEG data. Afterwards, a hybrid feature-set (Feature-Set 3) is formed by fusing feature-set 1 and 2. Phase two—the appropriate electrode site placement is selected, which has higher influence on stress detection rates. This part examines the electrode placements on different sites on the skull and selects the location which has the higher influence on the accuracy of the system. Phase three—we investigate the optimal number of principal components in the sequential forward search strategy to reduce the dimension of the feature space. Compressing feature space commonly improves the performance of an MSD system. Minimizing the number of EEG channels used in mental stress detection and evaluation would make the system more mobile and easier to set up, and maintain the real-time EEG-based mental stress detection system. Therefore, we come to phase four, where we examine the impact of each frontal electrode and select a minimum number of frontal electrodes in order to construct a portable MSD system. Five classifiers were built to identify stress and distinguish between two stress levels (low and high). Figure 3 illustrates the four experiments.

### 3.1. Experiment One Results

**Figure 4:**
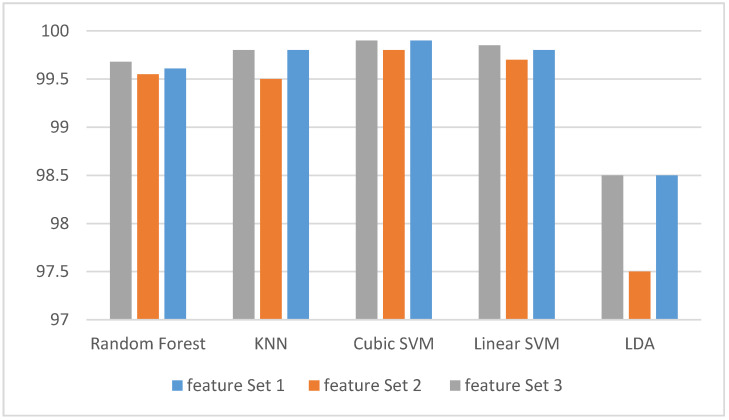
The classification accuracies for detecting stress and non-stress states for our three subsets of features.

**Figure 5:**
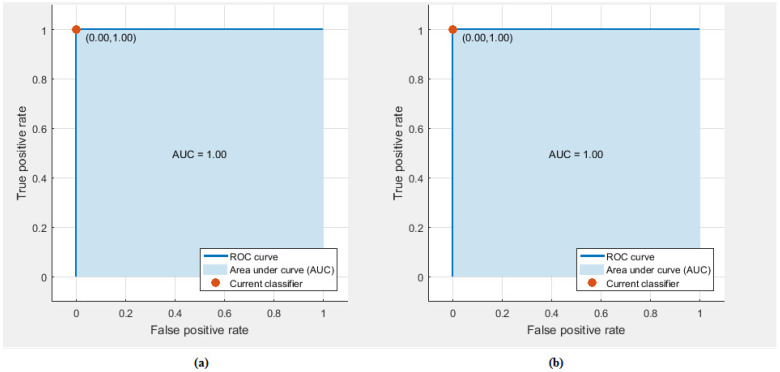
Receiver operating characteristic (ROC) curves for detecting stress and non stress; (**a**) cubic support vector machine (SVM) classifier, (**b**) *k*-nearest neighbour (KNN) classifer.

Two feature-sets (feature-set 1 and 2) were introduced in our study. Subsequently, a hybrid feature-set (Feature-Set 3) was made by combining these two feature-sets. These feature-sets were first used to detect stress and non-stress states. The classification accuracy of those three feature-sets, when used for detecting stress and non-stress states, are shown in Figure 4. It is clear from Figure 4 that Feature-Set 3 has higher accuracy than the other two feature-sets except for LDA which has the same performance as feature-set 1. Table 3 shows the evaluation metrics using Feature-Set 3 which achieved a higher accuracy for detecting stress. The sensitivity and specificity rates are all above 99% except for LDA, which is above 98%, and the sensitivity of linear SVM which is 98.84%. On the Goodness Index, they are all below 0.02. Figure 5 shows the ROC curve for detecting stress for both a cubic SVM and a KNN classifier. The area under ROC curve (AUC) is one.

**Figure 6:**
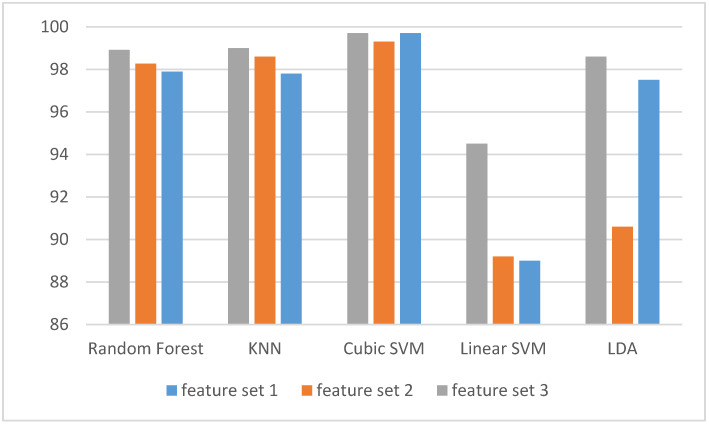
The classification accuracies for classifying stress levels using our three subsets of features.

**Figure 7:**
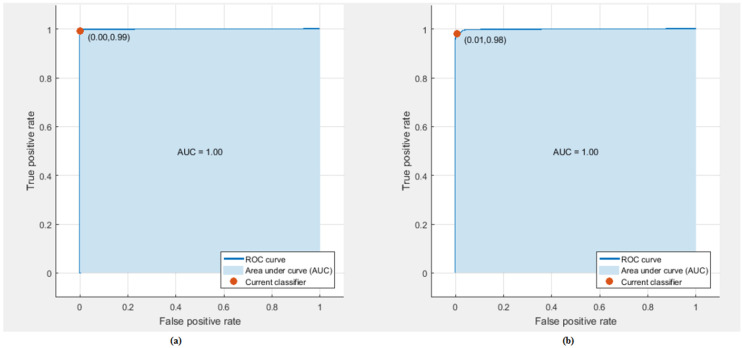
ROC curve for evaluating stress levels; (**a**) cubic SVM classifier, (**b**) KNN classifier.

Afterwards, the three subsets of features are used to classify stress levels. The classification accuracy of the three feature-sets used to classify stress levels is shown in Figure 6. Figure 6 shows that Feature-Set 3 yielded the highest accuracies using LDA, cubic and linear SVM, KNN and random forest classifiers respectively compared to the other two feature-sets. Table 4 shows the evaluation metric using Feature-Set 3 which achieved the highest accuracy for classification of stress levels. Table 3 indicates that the sensitivity ranges between (98%–100%) and the specificity ranges between (85%–99.4%). Figure 7 shows the ROC curve for detecting stress for both Cubic SVM and KNN classifier. The Goodness Index values are in the range of (0.00634–0.0151). The area under ROC curve (AUC) is one.

**Figure 8:**
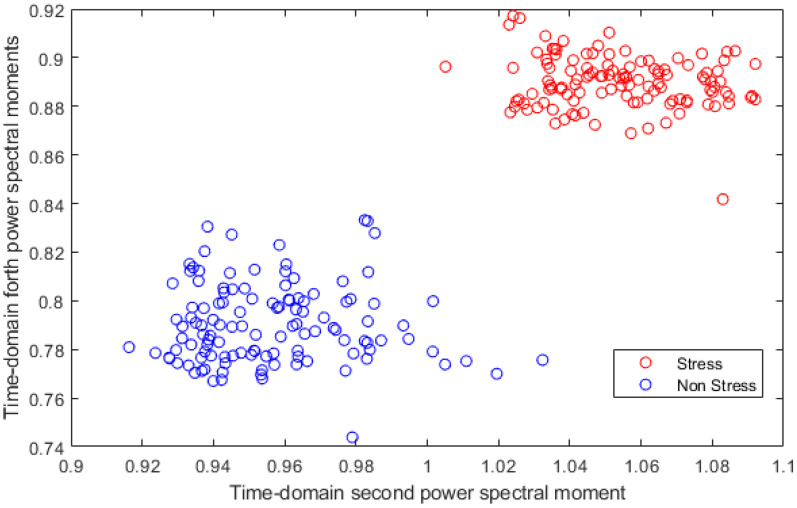
Two-dimensional scatter plot of time domain second power spectral moment vs. time domain forth power spectral moment feature for stress and non-stress cases.

**Figure 9:**
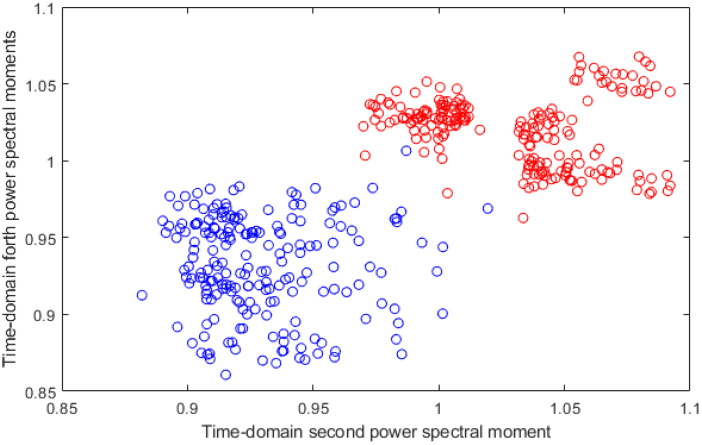
Two-dimensional scatter plot of time domain second power spectral moment vs. time domain forth power spectral moment feature for high- and low-stress level cases.

In order to show the ability of the proposed feature to distinguish between stress and non-stress scenarios and to separate between stress levels, two figures are plotted to present two-dimensional scatter plots of two different features of the proposed feature-set. Figure 8 shows a two-dimensional scatter plot of time domain second power spectral moment vs. time domain forth power spectral moment feature for stress and non-stress cases. Figure 9 shows a two-dimensional scatter plot of time domain second power spectral moment vs. time domain forth power spectral moment feature for high and low stress levels cases. These figures verify the effectiveness of the proposed features to differentiate between both stress and non-stress, and stress levels. 

### 3.2. Experiment Two Results

**Figure 10:**
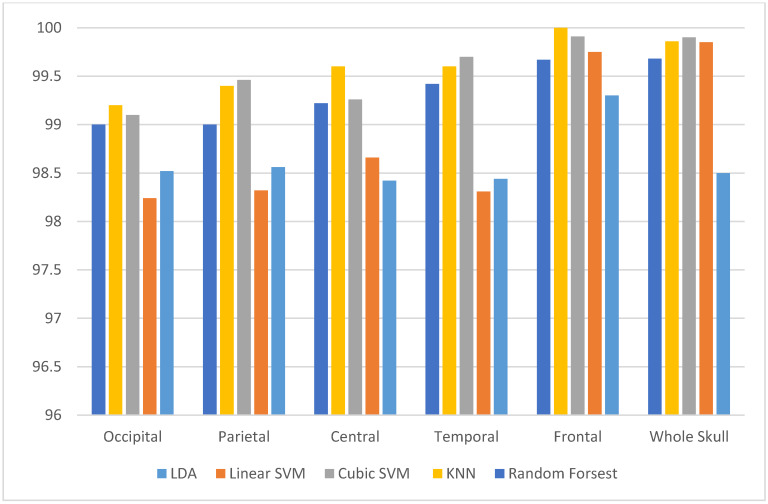
A comparison between classification accuracies of several classifiers constructed using the proposed Feature-Set 3 extracted from different sites on the skull to detect stress and non-stress states.

**Figure 11:**
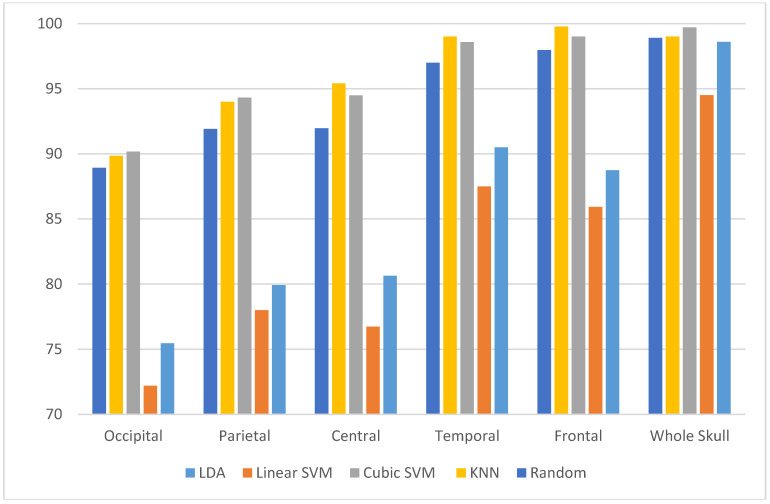
A comparison between classification accuracies of several classifiers constructed using the proposed Feature-Set 3 extracted from different sites on the skull to access stress levels.

As mentioned before, the main aim of the experiment is to explore the electrode locations on different sites on the brain and chooses the site which has the greater impact on the accuracy of the system. This process will also reduce the number of channels employed to construct the MSD system which accordingly makes it more reliable and efficient. Figure 10 shows a bar chart that compares between the performance of several classifiers constructed from Feature-Set 3 extracted from several sites on the skull to detect stress. It is clear from this figure that using only the frontal activation channel, the performance of the MSD system reached 99.98% using the KNN classifier which is higher than all other sites. Table 5 shows the values of accuracy for detecting stress and non-stress from different electrode sites. The frontal activation site has also greater impact on evaluating stress levels as well, shown in Figure 11. Figure 11 shows a bar chart that compares the performance of several classifiers constructed using the proposed Feature-Set 3 extracted from different sites on the skull to evaluate stress levels. The highest accuracy of 99.78% was achieved using the KNN classifier. Table 6 shows the values of accuracy for evaluating stress levels from different electrode sites

### 3.3. Experiment Three Results

**Figure 12:**
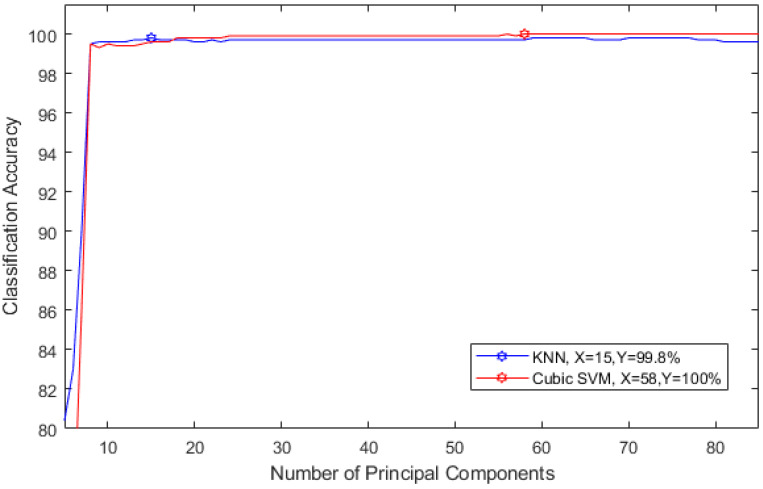
Selecting the optimal number of principle components for detecting stress.

The aim of this experiment is to investigate the influence of reducing the feature space using PCA on the performance of MSD system. The number of principal components are chosen in a sequential forward strategy. Figure 12 shows the selection process for the optimal number of principle components for detecting stress. Figure 12 shows that, using only 58 and 15 principal components, the accuracy of detecting stress reached 100% and 99.8% using cubic SVM and KNN classifiers respectively.

**Figure 13:**
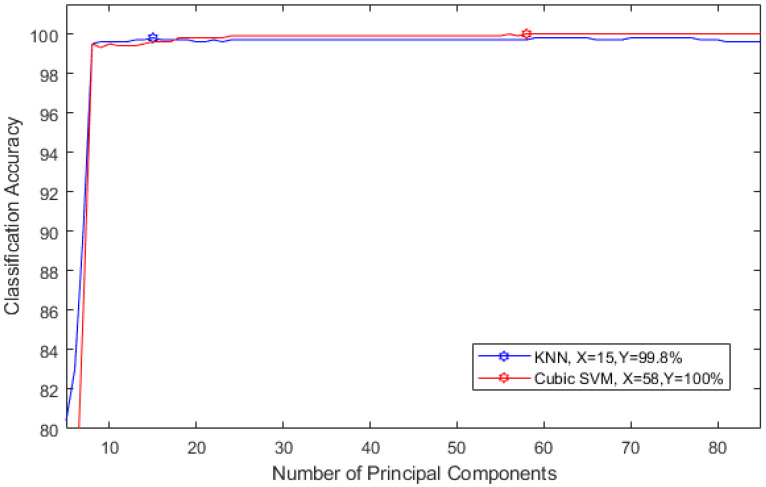
Selecting the optimal number of principle components for evaluating stress levels.

Figure 13 represents the selection procedure for the optimal number of principle components for evaluating stress levels. It is clear from Figure 13 that, using only 30 and 54 principal components, the accuracy of classifying stress levels reached 99.4% and 99.7% using cubic SVM and KNN classifiers respectively.

### 3.4. Experiment Four Results

As stated before, one of the aims of this manuscript is to build a portable real-time EEG-based mental training neuro-feedback system to detect and evaluate stress in real time with high accuracy. Minimizing the number of EEG electrodes used in mental stress detection and evaluation would make the system more portable and easier to operate, and maintain the real-time EEG-based mental stress detection system. In this experiment, the impact of each frontal electrode on stress detection and evaluation rates is investigated individually. Afterwards, the influence of fusing two or three electrodes which have higher detection and evaluation rates when used individually is examined for both stress detection and stress level evaluation. 

It is clear from Table 7 that Fp1 and Fp2 electrodes have the highest impact on detecting stress and non-stress. Therefore, each one can be used alone to detect stress, and the results show that only one frontal electrode, such as Fp1 or Fp2, is capable of detecting stress and non-stress. However, the impact of fusing Fp1 and Fp2 is examined and shown in Table 8, which shows the impact of using Fp1 and Fp2 on detecting stress and non-stress using five-fold cross validation. 

Table 8 shows that fusing Fp1 and Fp2 improves the performance metric for detecting stress, therefore the two Fp1 and Fp2 electrodes are sufficient to construct an efficient and portable MSD system. In order to further validate the performance of the proposed system and its ability to predict if a new person has stress or non-stress, leave-subject-out validation is performed as well and shown in Table 9. The results of Table 9 show the efficiency of the proposed system in predicting stress and non-stress on new patients.

In the case of evaluating stress levels, Table 7 shows that the highest accuracy achieved for evaluating stress levels using one electrode is 92.5% using the F8 electrode, followed by 92% and 91.36% using Fp1 and F7 respectively. To improve the performance of the proposed system for evaluating stress levels using the minimum number of electrodes, the impact of fusing two or three electrodes is investigated and shown in Table 10. 

The results of Table 10 show the ability of the proposed system to evaluate level increase using two electrodes. However, the performance of the proposed system is further improved using the three electrodes (Fp1 + F7 + F8). In order to further validate the performance of the proposed system and its ability to predict stress levels for a new person, leave-subject-out validation is performed as well and is shown in Table 11. However, the results of Table 11 show that the proposed system has a lower ability to predict stress levels.

## 4. Discussion

This study proposes an effective MSD system to detect stress and classify stress levels. For this purpose, the study design consists of three parts. The first part compares two feature-sets. The first feature-set consists of frequency-based features, whereas the second one consists of AR and RMS features. Here, the influence of combining time and frequency features is assessed. The analysis showed that combining time and frequency features increases stress detection and stress level classification rates. In order to verify the effectiveness of our proposed system, the results are compared with the classification accuracy of three feature-sets from literature. These include the feature extraction method proposed by Khushaba et al. [22] and the two approaches presented by Mahajan [18]. The two feature-sets reported by Mahajan include some peak related features and PSD features. Mahajan’s first feature-set comprises four peak-related features which are number of negative peaks, the number of positive peaks, the mean of negative peaks, and the mean of positive peaks. The second feature-set of Mahajan’s consists of the mean spectral power estimation in beta (13–30 Hz), alpha (8–13 Hz), theta (4–8 Hz), and delta (0.5–4 Hz) EEG sub-bands respectively. The feature-set of khushaba et al. represents features extracted using wavelet packet decomposition where the frequency components are selected using a mutual information estimation procedure that depends on the generalization of the fuzzy entropy theory.

**Figure 14:**
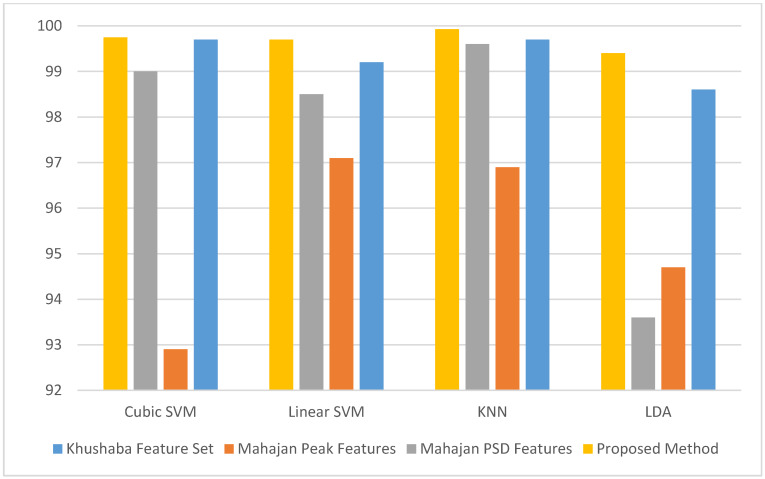
The classification accuracy of detecting stress and non-stress states using our proposed Feature-Set 3 compared to Khushaba et al. [22] and the two feature-sets from Mahajan [18] using only Fp1 + Fp2 frontal electrodes.

Figure 14 shows the accuracies for detecting stress and non-stress states using our proposed Feature-Set 3 compared to Khushaba and the two feature-sets from Mahajan using only Fp1 + Fp2 frontal electrodes. It is clear from Figure 14 that the accuracy of our proposed Feature-Set 3 (99.94%) is greater than that of Khashaba et al. (98.6%) and the two feature-sets of Mahajan (64.7%, 93.6%) using LDA classifier. Additionally, the accuracies of our proposed Feature-Set 3 (99.93%, 99.7, 99.75%) is greater than that of Khashaba et al. (99.6%, 99.2%, 99.7%), the peak feature-set of Mahajan (96.9%, 97.1%, 92.9%), and the PSD feature-set of Mahajan (99.6%, 98.5%, 99%) using KNN, Linear SVM, cubic SVM classifier respectively.

**Figure 15:**
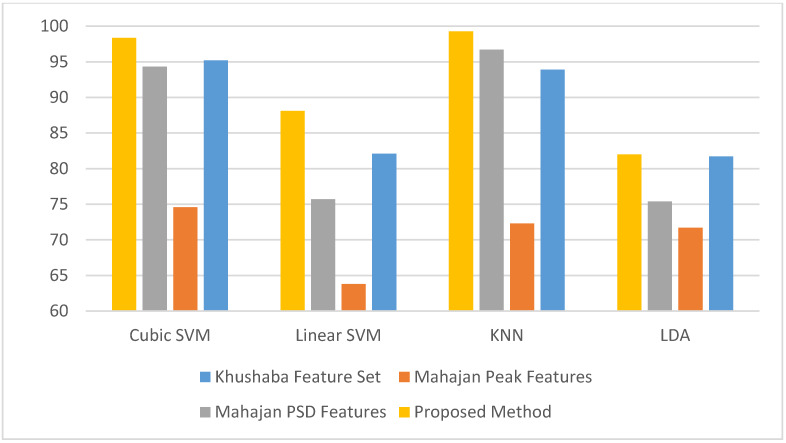
The classification accuracies for classifying stress levels using our proposed Feature-Set 3 compared to Khushaba et al. [22] and the two feature-sets from Mahajan [18].

The proposed Feature-Set 3 was also compared with Khushaba et al. and the two feature-sets from Mahajan, but in this case for evaluating stress levels using only Fp1 + F7 + F8 electrodes. Figure 15 shows the classification accuracies of classifying stress levels using our proposed Feature-Set 3 compared to Khushaba and the two feature-sets from Mahajan. It is clear from Figure 15 that the proposed Feature-Set 3 has better performance in evaluating stress levels compared to the other feature-sets from Khushaba et al. and Mahajan. the accuracy of our proposed Feature-Set 3 (82%, 99.26%, 88.1%, and 98.36%,) are higher than the Mahajan PSD feature-set (75.4%, 96.7%, 75.7%, and 94.3%), and Mahajan Peak feature-set (71.7%,72.3%, 63.8%, and 74.6%), using LDA, KNN, linear SVM, and cubic SVM classifiers respectively. The accuracy of the proposed Feature-Set 3 is also greater than Khushaba et al.’s feature-set as well (81.7%, 93.9%, 82.1%, and 95.2%), using LDA, KNN, linear SVM, and cubic SVM classifiers. This suggests that our proposed system, using the hybrid feature-set, is more efficient and has higher ability in identifying stress levels.

In the second part of the proposed technique, the selection of skull site and its influence on the performance of the MSD system are examined. Based on [4,30,31,55] that showed that EEG signals acquired from the frontal site of the skull are capable of evaluating and detecting the mental stress, this experiment was conducted and proved that frontal brain activation has the most impact on detecting and classifying stress levels. The accuracy of the proposed MSD with frontal brain activation reached 100% for detecting stress and 99.78% for classifying stress levels. The experiment showed that only seven electrodes are enough to achieve a reliable and efficient MSD system. Note that the feature space dimension is 228 after feature extraction. In the third part of the proposed study, PCA is used to reduce the feature space dimension and to construct the MSD and lower its complexity. Here, we showed that PCA is capable of improving the performance of MSD constructed using a cubic SVM classifier to reach an accuracy of 100% for detecting stress and 99.4% for classifying stress levels with only 58 and 54 principal components.

As it is clear from the results, KNN and SVM classifiers yielded the highest accuracies for both detections of stress and stress levels. This is because the KNN classifier is simple, straightforward and has high effectiveness, even with noisy datasets [56]. Despite its simplicity, it is able to produce high-accuracy rates in medical applications [57,58]. SVM is a strong classifier, and also has the capability to alter an input vector which is not linearly distinguishable using a hyperplane into a higher-dimensional feature space that is able to linearly discriminate between classes of input data to facilitate the classification process. The process is obtained using a kernel function which maps the likeness between the input data and the new higher-dimension feature space. A linear kernel is frequently used when the dataset is just divided by a linear line, which is the case in the feature space used in the case of detecting stress as shown in Figure 8. However, a quadratic kernel is a nonlinear kernel used when the dataset is complex and not linearly separable. Cubic kernels may possibly increase the accuracy. Other benefits of cubic kernels include taking sophisticated mathematical tractability and direct geometric interpretation [51]. The feature space in the case of classifying stress levels was not linearly separable, as shown in Figure 9; therefore, the cubic kernel produced better results than that of the linear kernel in classifying stress levels. 

EEG signals are commonly known to be non-stationary. This is due the changes in states of neuronal assemblies during brain functioning. It is essential to recognize non-stationaries in EEG signal, because they are illustrative of the underlying actions. This is done by segmenting the EEG signal into smaller stationary segments [59]. Selecting the appropriate window size which segments EEG signal into stationary segments is very important so that the model fits the actual and consistent activity of the brain [60]. There are several ways to check stationarity in literature. Among them, Azami et al. in [61] suggested that the standard deviation determined for each segment is can be a property that indicates changes in amplitude or/and frequency, as it remains unchanged in stationary intervals, the differences of the standard deviation in successive windows indicates stationarity. Furthermore, McEwen verified that short segments (up to 10 s) usually follow the normal distribution while longer segments (up to 60 s) are not Gaussian. McEwen recommended that EEG could be visualized as a procedure consisted from short Gaussian segments. A portion of segments that can be thought as Gaussian reduces from 90% to 20% when the segments’ period rises from 4 to 60 s. In contrast, up to 90% of 4-s-long segments can be believed as stationary while this number decreases to 70%–80% when analyzing 16 s-long EEG segments [2]. Therefore, we used 4-s segment length.

It was reported in [62,63] that for the medical system to be reliable, it should achieve a sensitivity greater than or equal to 80%, with a specificity greater than or equal to 95%, a precision greater than or equal 95%, and a DOR greater than or equal 100. The results in Table 8 show that the proposed system is reliable and can be used to detect stress and non-stress as the sensitivity is 99.9, specificity is 99.94, precision is 99.81, and DOR is 1057474 using a KNN classifier constructed with only Fp1 and Fp2 frontal electrodes. The results in Table 10 verify that the proposed system is reliable as well as capable of evaluating stress levels, as the sensitivity is 99.6, the specificity is 98.9, the precision is 98.15, and the DOR is greater than 100 using a KNN classifier constructed with only Fp1 + F7 + F8 frontal electrodes. This means that by using only one or two electrodes, the proposed system is capable of detecting stress and non-stress. It is also able to evaluate stress levels using only three frontal electrodes with high performances. The minimum number of electrodes selected to construct the proposed system makes the system more portable and mobile, and easier to set up.

Furthermore, to validate the ability of the proposed MSD system to predict if a new person has stress or non-stress, leave-subject-out validation is performed as well. The results in Table 9 confirm the capability of the proposed system in predicting stress and non-stress using only Fp1 and Fp2 frontal electrodes. They also reveal that the proposed system is reliable and can be used to predict stress and non-stress as the sensitivity is 97.78, the specificity is 97.75, the precision is 99.26, and the DOR is greater than 100 using a KNN classifier. Finally, the ability of the proposed MSD system to predict the stress level of a new person is tested using leave-subject-out validation. The results in Table 11 shows that the proposed system has lower capability in predicting stress levels. This is due the imbalance occurring between the two classes of stress levels. Therefore, future work will focus on investigating solutions to deal with this imbalance and improve the performance of predicting stress levels. 

Comparing the results of the proposed algorithm with recent related work in Table 1, it is quite clear that the proposed MSD system outperforms other recent related techniques from literature. More specifically, the accuracies achieved for the proposed technique are 99.9% (sd = 0.015) and 99.26% (sd = 0.08) for identifying stress and non-stress states, and distinguishing between stress levels, respectively, using only two frontal brain electrodes for detecting stress and non-stress, and three frontal electrodes for evaluating stress levels as shown in Table 8 and Table 10. The results prove that the proposed method has a competitive performance compared to the state-of-the-art techniques for both detecting stress and non-stress and classifying stress levels. 

## 5. Conclusions

The main aim of the proposed system is to build a portable real-time EEG-based mental training neuro-feedback system to detect and evaluate stress levels in real-time with high accuracy using mental arithmetic tasks. The proposed method introduced a hybrid feature-set (Feature-Set 3) and used five classification models for this purpose. Minimizing the number of EEG channels used in mental stress detection and evaluation would make the system more mobile and easier to set up, and maintain the real-time EEG-based mental stress detection system. This study revealed that the frontal brain activation has a great impact on detecting and evaluating stress levels and is capable of achieving high detection and classification rates. Additionally, the study indicated that PCA has the ability to reduce feature space and enhance stress detection rates. Furthermore, the study indicated that only one or two frontal electrodes are capable of detecting stress and non-stress, and three frontal electrodes are able to evaluate stress levels. The results showed that the proposed method based on the hybrid feature-set was capable of both identifying stress and classifying stress levels. Moreover, our method outperformed other feature extraction methods in literature. Furthermore, it has competitive performance compared to the state-of-the-art techniques. Thus, it can be used for stress management, industrial safety, and education. It will enable clinicians to make an accurate diagnosis and provide appropriate treatment, which will consequently reduce chances of clinical brain damage and other health problems. It will also improve the safety standards in industry and will enhance the quality of education. 

## Figures and Tables

**Table 1:** A summary of recent techniques from literature that are comparable to the proposed mental stress detection (MSD) system.

Reference	Number of Participants	Number of Electrodes	Class Label	Classifier	Validation Method	Accuracy
[25]	1	14	Stress and non-stress	Random Forest	k-fold cross validation	97.5%
[13]	50	14	Stress and non-stress	Threshold-based	Hold out	88%
[14]	7	31	Stress and non-stress	LDA	k-fold cross validation	82.6%
[19]	5	19	Stress and non-stress	ANN	Hold out	91.17%
[28]	4	22	Stress and non-stress	SVM	Hold out	78–79%
[11]	32	32	Stress and non-stress	KNN	k-fold cross validation	73.38%
[29]	32	32	Stress and non-stress	KNN	k-fold cross validation	71.76%
[31]	25	23	Stress and non-stress	SVM	k-fold cross validation	83.1%–89.8%
[30]	22	7	Stress and non-stress	SVM	Leave-one out	91.7%
[34]	27	5	Stress and non-stress	Linear Regression	k-fold cross validation	98.76%
[35]	50	16	Stress and non-stress	Ensemble	k-fold cross validation	97.95%
[26]	10	14	Stress levels	SVM	k-fold cross validation	96%
[20]	11	14	Stress levels	SVM	k-fold cross validation	80.32%
[27]	42	128	Stress levels	SVM	k-fold cross validation	94.6%
[10]	12	19	Stress levels	KNN	k-fold cross validation Hold out	91.5% 90.5%
[4]	20	1	Stress levels	SVM	k-fold cross validation	65%–75%
[34]	27	5	Stress Levels	Linear Regression	k-fold cross validation	95.062%
[36]	26	9	Stress Levels	KNN	Leave one out	90.9%
[37]	10	4	Stress Levels	LDA	Leave-one-out	86%
[38]	12	7	Stress Levels	SVM	k-fold cross validation	80%–85%
[39]	28	1	Stress Levels	SVM	k-fold cross validation	78.57%

**Table 2:** Details of the participants.

Name	Age (Years)	Gender
Participant 1	21	Female
Participant 2	18	Female
Participant 3	19	Female
Participant 4	17	Female
Participant 5	17	Female
Participant 6	16	Female
Participant 7	18	Male
Participant 8	18	Female
Participant 9	26	Male
Participant 10	16	Female
Participant 11	17	Female
Participant 12	18	Female
Participant 13	17	Female
Participant 14	24	Male
Participant 15	17	Female
Participant 16	17	Female
Participant 17	17	Female
Participant 18	17	Female
Participant 19	17	Female
Participant 20	22	Male
Participant 21	17	Female
Participant 22	19	Female
Participant 23	20	Female
Participant 24	16	Female
Participant 25	17	Male
Participant 26	17	Male
Participant 27	17	Female
Participant 28	19	Female
Participant 29	19	Female
Participant 30	19	Male
Participant 31	17	Male
Participant 32	19	Female
Participant 33	20	Female
Participant 34	17	Male
Participant 35	18	Female
Participant 36	17	Female

**Table 3:** Evaluation metrics (with their 95% confidence intervals) for detecting stress using Feature-Set 3.

Classifier	Accuracy (%)	Sensitivity (%)	Specificity (%)	Goodness Index	Precision (%)	MCC (%)	DOR
LDA	98.50 (98.49–8.63)	98.89 (98.85–98.93)	98.45 (98.17–98.73)	0.0198 (0.0196–0.020)	99.83 (99.69–99.96)	99.7 (99.66–99.76)	1.3 × 10^6^ (0.669–1.952) × 10^6^
Linear SVM	99.85 (99.8–99.88)	98.84 (99.81–9.876)	99.86 (99.85–99.88)	0.020 (0.0017–0.002)	99.95 (99.94–99.95)	99.64 (99.57–99.7)	1.36 × 10^6^ (0.504–3.2306) × 10^6^
Cubic SVM	99.90 (99.85–99.93)	99.88 (99.81–99.95)	99.9 (99.88–99.93)	0.015 (0.0009–0.002)	99.96 (99.94–99.96)	99.7 (99.67–99.74)	8.08 × 10^5^ (6.209–9.946) × 10^5^
KNN	99.86 (99.84–99.89)	99.95 (99.91–99.99)	99.79 (99.75–99.81)	0.0022 (0.002–0.0025)	99.99 (99.98–100)	99.64 (99.94–99.98)	>1000
Random Forest	99.68 (99.64–99.72)	99.52 (99.42–99.62)	99.76 (99.69–99.83)	0.0054 (0.0044–0.006)	99.8 (99.8–99.8)	99.14 (99.08–99.19)	7.8 × 10^4^ (6.8783–8.733) × 10^4^

**Table 4:** Evaluation metrics (with their 95% confidence intervals) for evaluating stress levels using Feature-Set 3.

Classifier	Accuracy (%)	Sensitivity (%)	Specificity (%)	Goodness Index	Precision (%)	MCC (%)	DOR
LDA	98.6 (98.37–98.83)	99 (98.85–99.56)	97 (96.32–97.60)	0.03156 (0.0259–0.037)	97.98 (97.68–98.27)	96.63 (96.25–97)	4589.8 (3451–5728.6)
Linear SVM	94.5 (94.08–94.84)	98 (97.83–98.24)	85 (83.93–86.11)	0.151 (0.1401–0.161)	94.16 (93.55–94.77)	85.42 (85.19–85.71)	271.2 (244.74–297.65)
Cubic SVM	99.7 (99.52–99.79)	99.85 (99.82–99.89)	99.4 (99.05–99.73)	0.00634 (0.0032–0.009)	99.69 (99.61–99.76)	99.49 (99.37–99.62)	2.39 × 10^5^ (1.255–3.53) × 10^5^
KNN	99 (98.86–99.03)	99.4 (99.25–99.47)	97.7 (97.49–98.10)	0.02296 (0.020–0.0259)	98.27 (97.88–98.66)	97.27 (97.03–97.51)	6.97 × 10^3^ (5.426–8.50) × 10^3^
Random Forest	98.91 (98.77–99.05)	100 (100–100)	96.02 (95.44–96.59)	0.0398 (0.034–0.0456)	100 (100–100	97.38 (97.19–97.56)	>1000

**Table 5:** Accuracy (95% CI) for detecting stress and non-stress from different electrode sites.

Classifier	Frontal	Temporal	Central	Parietal	Occipital
LDA	99.3 (99.28–99.32)	98.44 (98.37–98.50)	98.42 (98.36–98.48)	98.56 (98.45–98.67)	98.52 (98.46–98.58)
Linear SVM	99.75 (99.67–99.82)	98.31 (98.26–98.38)	98.66 (98.14–99.18)	98.32 (98.18–98.46)	98.24 (98.17–98.30)
Cubic SVM	99.91 (99.88–99.93)	99.7 (99.67–99.73)	99.26 (99.19–99.33)	99.46 (99.39–99.53)	99 (98.92–99.12)
KNN	99.98 (99.96–100)	99.6 (99.81–99.92)	99.6 (99.56–99.67)	99.4 (99.245–99.55)	99.2 (99.05–99.35)
Random Forest	99.67 (99.65–99.69)	99.42 (99.39–99.46)	99.22 (99.19–99.25)	99 (98.97–99.11)	99 (98.96–99.08)

**Table 6:** Accuracy (95% CI) for evaluating stress levels from different electrode sites.

Classifier	Frontal	Temporal	Central	Parietal	Occipital
LDA	88.74 (88.30–89.18)	90.5 (90.11–90.89)	80.64 (80.39–80.88)	79.92 (79.59–80.05)	75.46 (75.29–75.63)
Linear SVM	85.92 (85.34–86.49)	87.5 (87.19–87.76)	76.74 (76.43–77.04)	78 (77.79–78.25)	72.2 (72.2–72.2)
Cubic SVM	99 (98.79–99.24)	98.58 (98.31- 98.85)	94.48 (93.96–95.0)	93.32 (92.83–93.80)	90.18 (89.82– 90.53)
KNN	99.78 (99.72–99.84)	99 (98.88–99.12)	95.4 (94.96–95.84)	94 (93.81–94.27)	89.84 (89.64–90.03)
Random Forest	97.91 (97.73–98.09)	97 (96.73–97.33)	91.96 (91.69–92.22)	91.92 (91.73–92.12)	88.93 (88.64–89.22)

**Table 7:** Accuracy (%) with (95% CI) for detecting stress and non-stress, and evaluating stress levels using each frontal electrode individually using five-folds cross validation.

Classifier	Fp1	Fp2	F3	F4	FZ	F7	F8
**Detection of Stress and Non-stress**							
KNN	99.74 (99.70–99.78)	99.54 (99.49–99.59)	99.37 (99.35–99.39)	97.82 (97.77–97.86)	99 (98.98–99.08)	99.29 (99.21–99.37)	98.87 (98.93–99.02)
Cubic SVM	99.64 (99.63–99.65)	99.39 (99.35–99.43)	99.18 (99.14–99.22)	97.78 (97.73–97.84)	99.22 (99.20–99.25	99.14 (99.09–99.19)	99 (98.99–99.07)
**Evaluating Stress Levels**							
KNN	92 (91.71–92.29)	90 (89.82–90.34)	90.74 (90.32–91.16)	85.58 (85.32–85.85)	88.76 (87.96–89.56)	91.36 (90.92–91.79)	92.5 (92.18–92.82)
Cubic SVM	88.48 (88.06–88.89)	88.48 (88.21–88.75)	88.62 (88.07–89.17)	87.3 (87.0–87.59)	89.58 (89.29–89.87)	92.26 (91.89–92.63)	90.98 (90.60–91.36)

**Table 8:** Performance metrics (95% CI) for detecting stress and non- stress with Fp1 and Fp2 electrodes using five-folds cross validation.

Classifier	Accuracy	Sensitivity	Specificity	Precision	MCC	Goodness Index	DOR
KNN	99.9 (99.84–99.97)	99.9 (99.65–99.99)	99.94 (99.84–99.98)	99.81 (99.49–99.93)	99.74 (99.71–99.77)	0.006 (0.004–0.007)	1057474 (0.67–1.44) × 10^6^
Cubic SVM	99.75 (99.74–99.77)	99.65 (99.58–99.71)	99.79 (99.77–99.81)	99.37 (99.31–99.43)	99.34 (99.29–99.40)	0.0041 (0.003–0.005)	124250 (1.14–1.35) × 10^5^

**Table 9:** Performance metrics (95% CI) for predicting stress and non-stress with Fp1 and Fp2 electrodes using leave-subjects-out cross validation.

Classifier	Accuracy (%)	Sensitivity (%)	Specificity (%)	Precision (%)	MCC (%)	Goodness Index	DOR
KNN	98.48 (97.47–99.49)	97.78 (97.07–100)	97.75 (95.90–99.59)	99.26 (98.66–99.86)	96.14 (93.69–98.58)	0.032 (0.0041–0.05)	>1000
Cubic SVM	98.67 (97.82–99.53)	98.96 (97.67–100)	98.26 (95.68–100)	99.29 (98.44- 100)	96.6 (94.47–98.73)	0.02 (0–0.04)	>1000

**Table 10:** Performance metrics (95% CI) of the proposed system constructed using (Fp1 + F7) and (Fp1 + F7 + F8) to evaluate stress levels using five-folds cross validation.

Classifier	Accuracy (%)	Sensitivity (%)	Specificity (%)	Precision (%)	MCC (%)	Goodness Index	DOR
**Fp1 + F8**							
KNN	97.52 (94.68–100)	97.59 (97.19–97.97)	99.36 (99.15–99.57)	98.37 (97.9–98.83)	97.32 (96.94–97.69)	0.025 (0.021–0.096)	7708 (0.51–1.03) × 10^4^
Cubic SVM	97.48 (97.37–97.58)	94.96 (94.6–95.27)	98.46 (98.37–98.54)	95.56 (95.75–96.16)	93.73 (93.47–93.97)	0.053 (0.049–0.056)	3611 (1.56–8.78) × 10^3^
**Fp1 + F7 + F8**							
KNN	99.26 (99.17–99.34)	98.35 (98.05–98.64)	99.6 (99.56–99.66)	98.9 (98.65–99.15)	98.15 (97.99–98.32)	0.017 (0.014–0.02)	15935 (1.37–1.81) × 10^4^
Cubic SVM	98.36 (98.25–98.46)	96.65 (96.23–97.07)	99 (98.89–99.15)	97.43 (97.10–97.75)	95.89 (95.62–96.15)	0.035 (0.031–0.04)	2977 (2.6–3.34) × 10^3^

**Table 11:** Performance metrics for predicting stress levels with Fp1 + F8 and Fp1 + F7 + F8 electrodes using leave-subjects-out cross validation.

Classifier	Accuracy (%)	Sensitivity (%)	Specificity (%)	Precision (%)	MCC (%)	Goodness Index	DOR
**Fp1 + F8**							
KNN	76.85 (67.14–86.58)	67.15 (53.17–81.25)	78.85 (64.54–93.15)	63.83 (49.6–77.56)	48.88 (34.13–63.63)	0.39 (0.2–0.58)	17 (7.669–26.4)
Cubic SVM	75.8 (66.59–84.99)	65.26 (50.37–76.14)	80.3 (65.89–94.73)	61.64 (47.85–75.42)	44.87 (30.79–58.95)	0.4 (0.24–0.61)	14.13 (2.675–25.59)
**Fp1 + F7 + F8**							
KNN	76.2 (65.77–86.61)	65.66 (48.56–82.77)	80.14 (64.02–96.256	63.48 (47.97–78.99)	47.85 (32.13–63.57)	0.444 (0.295–0.593)	22.28 (0.6879–43.87)
Cubic SVM	73.83 (62.82–84.83)	52.04 (34.86–69.22)	77 (58.98–95.13)	60.77 (44.26–77.28)	48 (32.25–63.81)	0.545 (0.424–0.67)	12.39 (3.92–20.86)
